# Application of Hyperbaric Oxygen Therapy (HBOT) as a Healing Aid after Extraction of Incisors in the Equine Odontoclastic Tooth Resorption and Hypercementosis Syndrome

**DOI:** 10.3390/vetsci9010030

**Published:** 2022-01-15

**Authors:** Kamil Górski, Elżbieta Stefanik, Andrzej Bereznowski, Izabela Polkowska, Bernard Turek

**Affiliations:** 1 Department of Large Animal Diseases and Clinic, Institute of Veterinary Medicine, Warsaw University of Life Sciences, Nowoursynowska 100, 02-797 Warsaw, Poland; bernard_turek@sggw.edu.pl; 2 Dierenkliniek Equinox, Vekemans 10, 2460 Kasterlee, Belgium; 3 Department of Veterinary Epidemiology and Economics, Faculty of Veterinary Medicine, Warsaw University of Life Sciences, 02-787 Warsaw, Poland; andrzej_bereznowski@sggw.edu.pl; 4 Department and Clinic of Animal Surgery, Faculty of Veterinary Medicine, University of Life Sciences in Lublin, 20-612 Lublin, Poland; izabela.polkowska@up.lublin.pl

**Keywords:** hyperbaric oxygen therapy, equine odontoclastic tooth resorption and hypercementosis syndrome, incisors extraction, equine dentistry, veterinary dentistry, oral surgery

## Abstract

Hyperbaric Oxygen Therapy (HBOT) is a stress-free, relatively safe method supporting the treatment of many different diseases. Although it is widely used in human medicine (including dentistry), in veterinary medicine, especially in the treatment of horses, there are not many scientifically described and documented cases of its use. Equine Odontoclastic Tooth Resorption and Hypercementosis syndrome is a disease that affects older horses and significantly reduces their quality of life. The only effective treatment for this condition is extraction of the incisors. The described case compares the recovery process of the alveolar area after extraction of incisors during the course of EOTRH syndrome without and with the use of a chamber, in horses with the same clinical picture of the disease, of the same age, and living in the same conditions. According to the authors’ knowledge, the presented case describes the use of a chamber in equine dentistry for the first time.

## 1. Introduction

Hyperbaric oxygen therapy (HBOT) is a safe, stress-free method that can be used as an adjunct therapy for a wide variety of conditions. This method involves administering an increased concentration of oxygen at a pressure higher than ambient, which allows the concentration of oxygen dissolved in the plasma to be multiplied [1,2,3,4]. As a result, the increased oxygen concentration reaches the diseased tissues, even those with poor blood supply [2]. Among other things, this supports the aerobic mechanisms of action of neutrophils killing anaerobic bacteria [1,3]. HBOT stimulates the angiogenesis process, reduces tissue swelling, and stimulates the proliferation of fibroblasts [5]. This allows for the acceleration of wound healing and shortens the recovery time after surgical procedures. Despite the fact that there are centers in the world where HBOT is used, there are not many literature reports on its effectiveness. This therapy is well-documented in human medicine, including human dentistry, where the process of treating single cases is most often depicted, mainly for difficult-to-heal wounds [2,4,5,6,7,8]. According to the authors’ knowledge, this is a description of the first use of HBOT in equine dentistry, therefore our observation was performed as a pilot study. Moreover, the normal healing process compared to assisted healing with HBOT has not yet been documented.

Equine Odontoclastic Tooth Resorption and Hypercementosis syndrome (EOTRH) is a painful, progressive disease that affects older horses (over 15 years of age) [9]. Clinical signs include difficulties during prehension, especially hard fruits and vegetables, ptyalism, halitosis, oral pain, furcation exposure and gingival recession, gingivitis, excessive calculus deposition, receding gumlines, prominent juga, periodontal fistulation, bulbous, cauliflower like incisors, or fractured or loose teeth. The clinical symptoms of the disease largely depend on which of the coexisting processes plays the dominant role—odontoclastic tooth resorption or hypercementosis [10]. This disease is diagnosed in horses all over the world, and the only effective method of its treatment is the extraction of all the affected teeth. In the described cases, we compared the process of alveolar area recovery after the extraction of incisors in two horses with the same disease picture, of the same age, and living in the same conditions, in which one of them underwent hyperbaric oxygen therapy and the other did not. The obtained results relate only to the visual observation of the treatment progress. In this study, due to the nature and location of the wound, it was impossible to carry out objective measurement methods such as thickness of the skin layer, wound surface area, or the activity of the sweat glands.

## 2. Materials and Methods

### 2.1. Case Presentation

#### 2.1.1. Case 1

**Figure 1:**
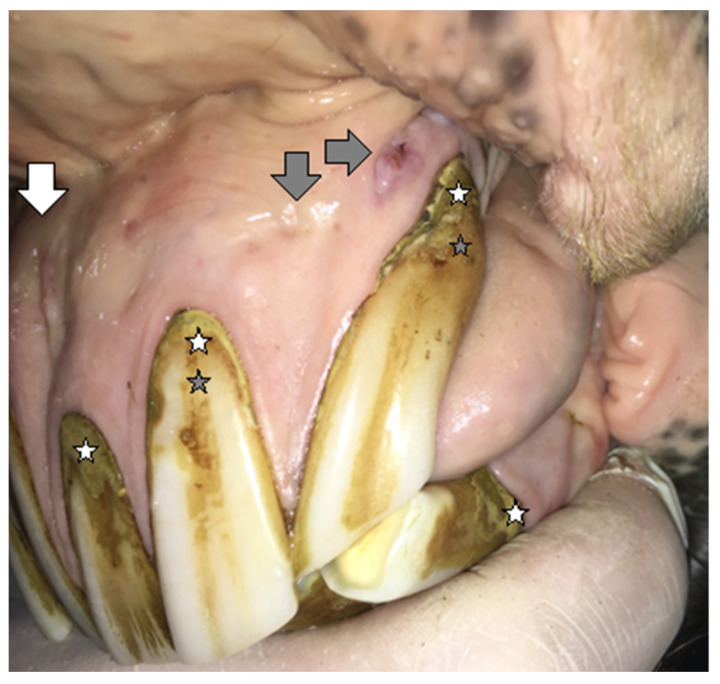
Case 1—The condition of maxillary incisors before extraction. Deformation of the incisal area, a depressions between the ridges of bone formed by roots in the alveolar process on the maxilla, called prominent juga (white arrow). Excessive calculus deposition (white stars), periodontic fistulas (gray arrows), and recessing gumlines (gray stars).

**Figure 2:**
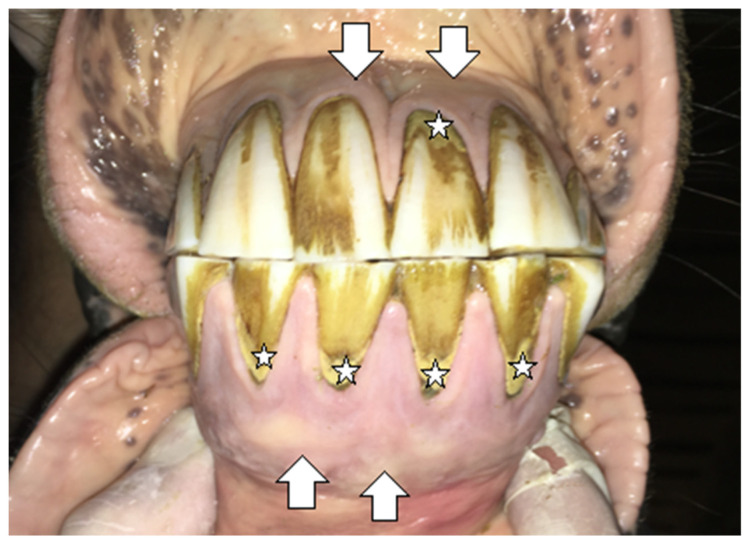
Case 1—the condition of the maxillary and mandibular incisors before extraction. Deformation of the incisal area (white arrows). Excessive calculus deposition (white stars).

**Figure 3:**
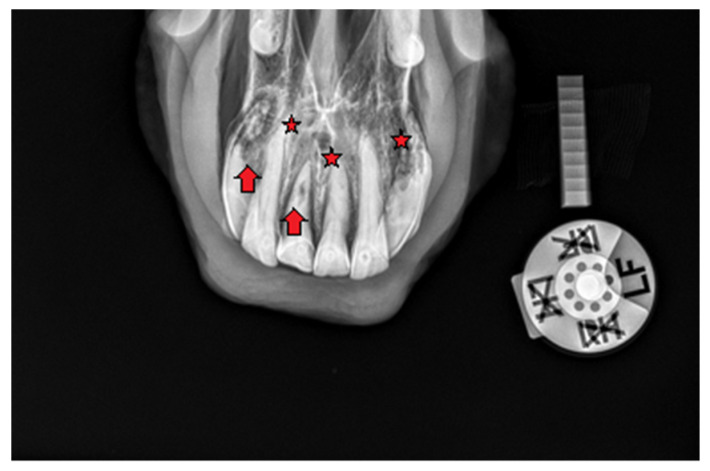
Case 1—intraoral radiograph of maxillary incisor teeth. Red arrows show areas of extensive resorption. Osteomyelitis of maxilla (red stars).

**Figure 4:**
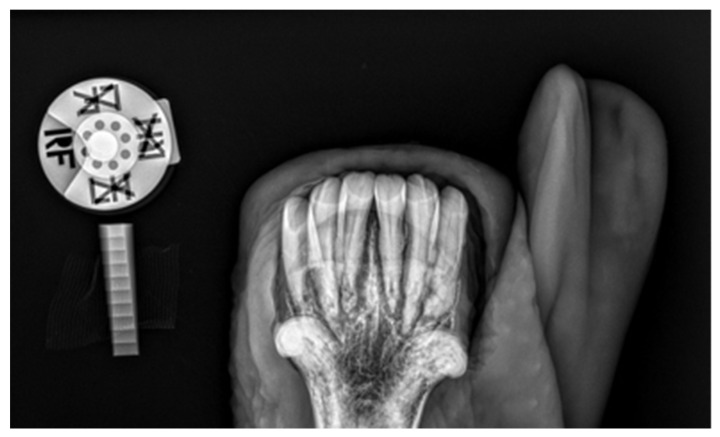
Case 1—intraoral radiograph of mandibular incisor teeth. Radiolucent areas around the tooth roots, indicating extensive resorption, erosion of the apical part of the root, irregular and rough surface of an intra-alveolar part.

**Figure 5:**
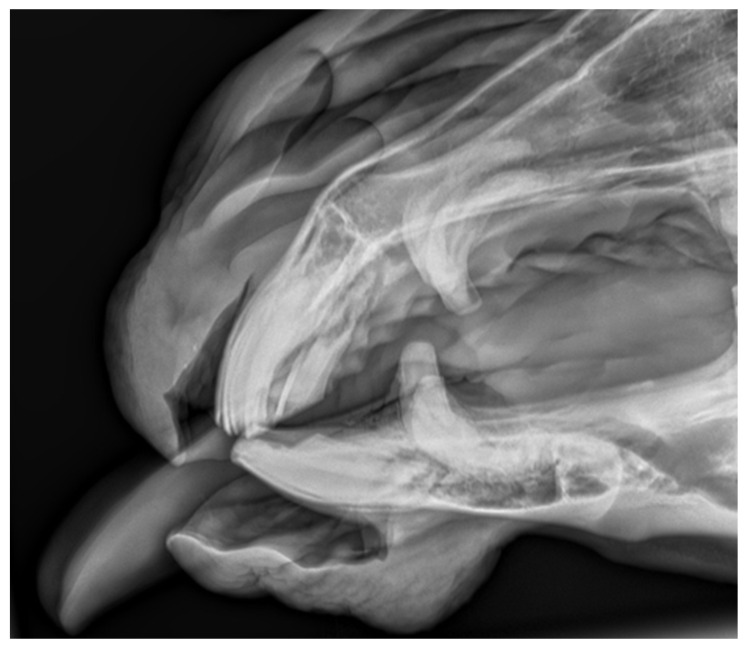
Case 1—lateral projection of the incisal area.

A 20-year-old Polish Half-Bred gelding weighing 650 kg used for recreational purposes was subjected to an oral examination, during which the EOTRH syndrome was found on the basis of the clinical examination (Figure 1 and Figure 2). At the time of the horse’s examination, all general parameters were within physiological limits. The condition and maintenance of the horse was appropriate for its age and level of work. It is known from the medical history that the examination of the oral cavity and the correction of teeth in this horse were performed regularly, approximately every 12 months, although there is no detailed information about the possible correction of the incisors. EOTRH syndrome was not diagnosed in the previous years. During detailed examination of the incisal area of the mouth, the presence of a ventral curvature was found, created by the contact of the rubbing surfaces of the incisors of the maxilla and mandible. Compression of the outermost incisors of the maxilla and the mandible caused pain and the receding of the head as a defensive reaction of the animal. In addition, gingivitis on the labial and lingual surfaces of all incisors was found, as well as a clear gingival recession around teeth 101, 102, 103, 201, 202, 203, 303 and 403 according to the Modified Triadan system. Deformation of the labial surface in the area of the incisal processes was found on all incisors. Fistula openings were also present, located on the labial surface of the roots and reserve crowns of teeth 101, 102, 103, 201, 202, 203, 303, 401, 402, 403. Deposition of dental calculus was identified on the labial surface of teeth 101, 102, 201, 202, 203 and all mandibular incisors (especially corners). To confirm the EOTRH Syndrome, a radiological examination of the area of the incisal processes was performed in the following intraoral projections—dorsoventral (Figure 3) for the maxillary incisors and ventrodorsal (Figure 4) for the mandibular incisors. Additionally, a lateral projection of the incisal area was performed (Figure 5). On the basis of the obtained image, decreased opacity of the roots and reserve crown areas of the incisors, as well as lamina dura of the alveolar processes of maxillary incisors (especially the extreme ones) and of the mandibular incisors was found. Radiographs confirmed the presence of the advanced EOTRH Syndrome, with a predominance of the resorptive process. The process of excessive cement deposition was most evident in the area of the root and reserve crown of tooth 303. Further examination of the oral cavity was performed after thorough rinsing with water and application of a Hausmann speculum. A light source, dental mirror, and dental probe were then used to examine individual teeth. The examination of the oral cavity revealed calculus deposition and gingivitis with the simultaneous recessing of teeth 304 and 404, sharp edges of the cheek teeth in the maxilla and mandible, erosions of the cheek and tongue mucosa, and excessively protruding teeth 306 and 406. In the complete blood count, red and white blood cell parameters were within physiological limits, with the exception of slight fluctuations in individual parameters: lymphocytes—below normal (0.7 × 10^9^/L), granulocytes—above normal (78.8%), hemoglobin—below normal (102 g/L).

#### 2.1.2. Case 2

**Figure 6:**
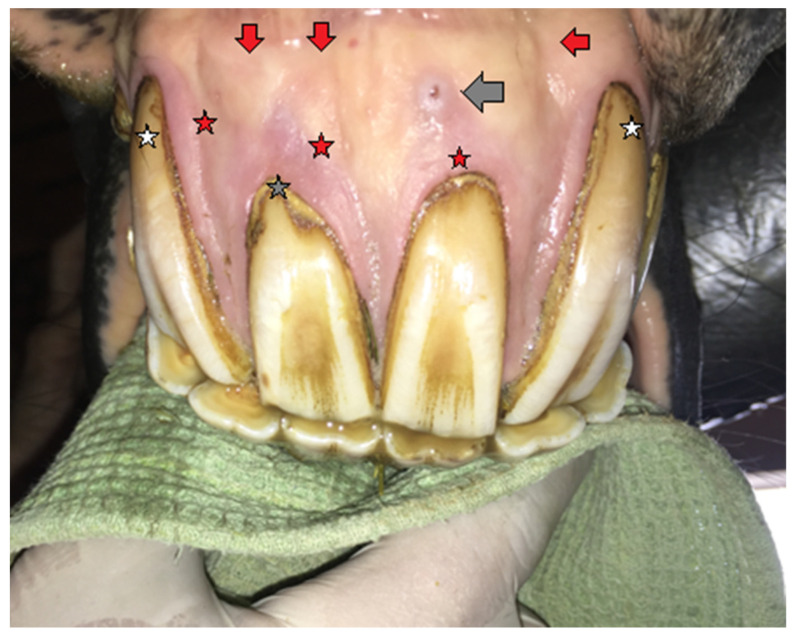
Case 2—the condition of the maxillary incisors before extraction. White stars—recessing gumlines, gray stars—excessive calculus deposition, red stars—gingivitis, gray arrow—fistula, red arrows—deformation of incisal processes.

**Figure 7:**
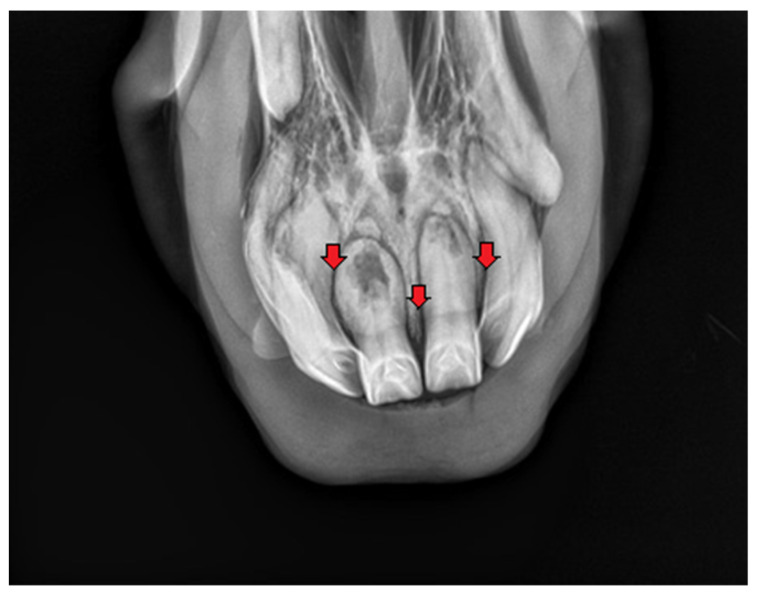
Case 2—intraoral radiograph of maxillary incisor teeth. Bulbous enlargement of the intra-alveolar part of the teeth, widening of periodontal ligament space (red arrows).

**Figure 8:**
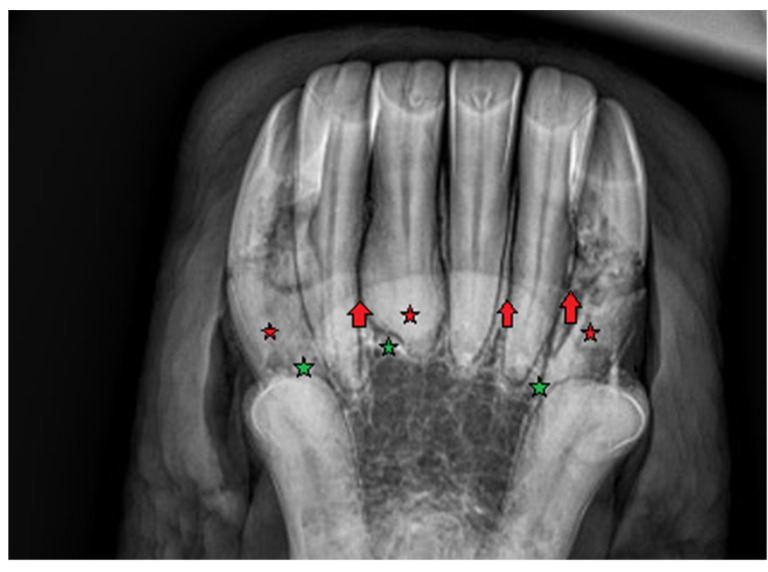
Case 2—intraoral radiograph of mandibular incisor teeth. Bulbous enlargement of the intra-alveolar part of the teeth (red stars), widening of periodontal ligament space (red arrows), lytic appearance of roots (resorption), disruption of *lamina dura* (green stars).

**Figure 9:**
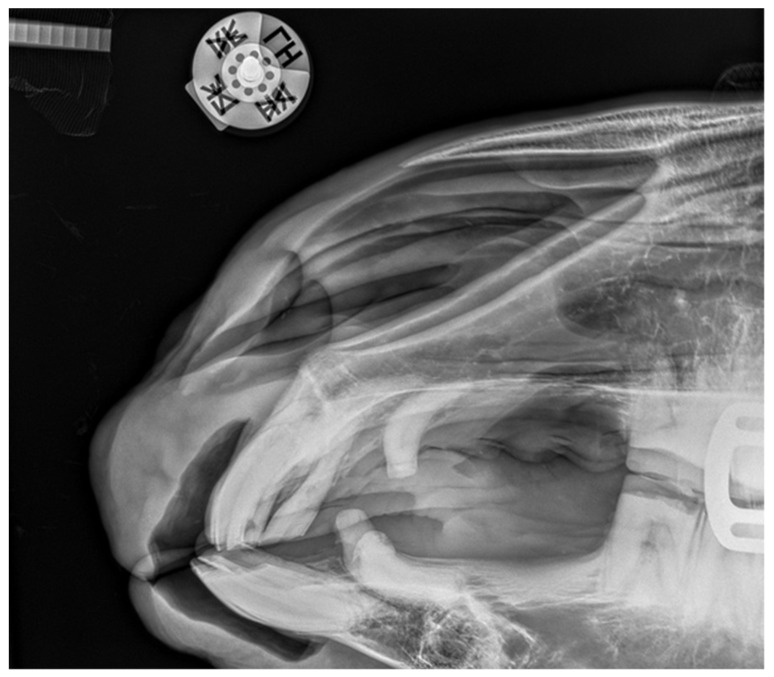
Case 2—lateral projection of the incisal area. Visible deformation of the incisal processes and osteomyelitis.

EOTRH syndrome was diagnosed in a 21-year-old Oldenburg gelding with a body weight of 700 kg and used for recreational purposes. It is known from medical history that the oral cavity inspection was performed regularly, every 12 months. If necessary, the dentition was corrected, consisting of the removal of sharp edges of the cheek teeth in the maxilla and mandible. There is no information on the possible correction of the incisors. The presented horse has never manifested any major health problems. As in case 1, EOTRH Syndrome was not previously diagnosed. At the time of the horse’s examination, all general parameters were within physiological limits. The condition and maintenance of the horse were assessed as appropriate. Detailed examination of the incisal area of the mouth showed the presence of a ventral curvature and a slight undershot bite. Compression of the corner incisors showed a painful reaction. Tooth 102 was missing. Gingivitis was recognized around teeth 103, 303 and 403. Gingival recession was found around teeth 101, 103, 201, 202, 203 and all mandibular incisors. In addition, the presence of closed diastema between teeth 202 and 203 was recognized, with a tendency to accumulate food content within it. Calculus deposition was found in the area of tooth 203. As in case 1, deformation of the labial surface in the area of the alveolar processes was found in all of the incisors present. A single fistula opening was located on the labial surface of the root area of tooth 201. Based on a detailed examination of the area of the incisal processes, the diagnosis of EOTRH Syndrome was made (Figure 6). The radiographs of the incisal area were made in the same way as in the case of 1. The following changes were recognized: decreased opacity of the apical part of the root, significant widening of the periodontal ligament space (especially within the central incisors of the maxilla and mandible), root resorption (especially the extreme mandibular incisors), extensive hypercementosis of the reserve crowns and roots of all incisors, loss of lamina dura osteitis of the alveolar processes of the maxilla and mandible (Figure 7, Figure 8 and Figure 9). Examination of the rest of the oral cavity revealed calculus deposition and gingivitis with simultaneous gingival recession of teeth 304 and 404, sharp enamel points on the buccal side of the maxillary cheek teeth and lingual side of the mandibular cheek teeth, erosions of the cheek and tongue mucosa, excessively protruding teeth 306 and 406, and inactive infundibular caries of teeth 109 and 209. The blood count results were within acceptable standards.

### 2.2. Treatment and Postoperative Care

**Figure 10:**
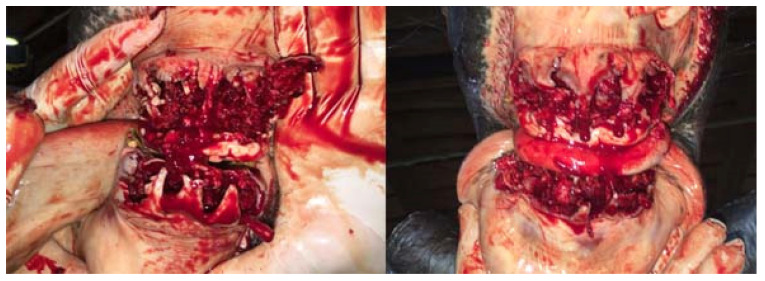
The condition of the tooth extraction sockets immediately after the extraction (on the left—case 1, on the right—case 2).

In both cases, based on the results of a detailed clinical and radiological examination and the expected exacerbation of the disease, a decision was made to extract all incisors (Figure 10). Before the incisor extraction procedure, the required correction of the canines and cheek teeth was performed in both horses, consisting of the mechanical removal of calculus deposition from the canines, their slight rounding, removal of sharp edges of the cheek teeth in the mandible and shortening of the clinical crown of excessively protruding teeth.

**Figure 11:**
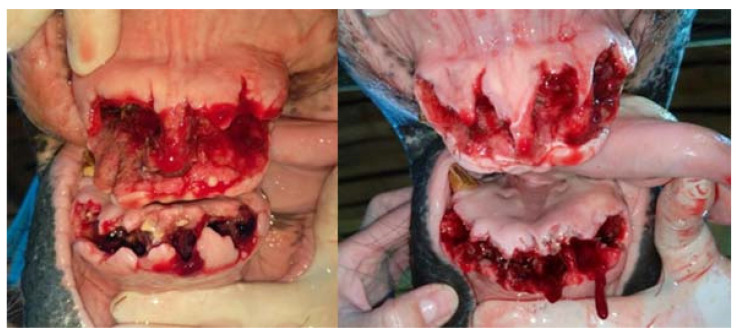
The condition of the tooth extraction sockets during the first change of alveolar dressings 4 days after the procedure (on the left—case 1, on the right—case 2).

**Figure 12:**
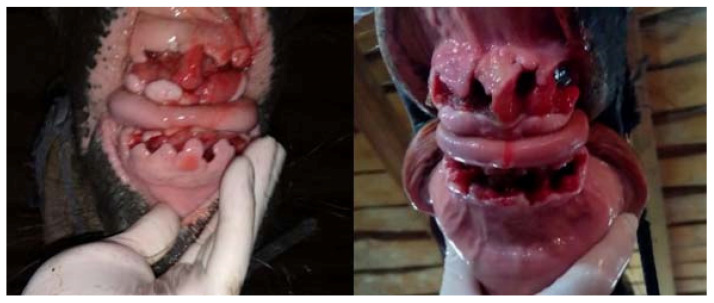
The condition of the tooth extraction sockets during the first change of alveolar dressings 9 days after the procedure (on the left—case 1, on the right—case 2). The horse undergoing hyperbaric oxygen therapy (case 1) had less swelling, faster gingival shrinkage and faster filling of the alveoli with granulation tissue.

**Figure 13:**
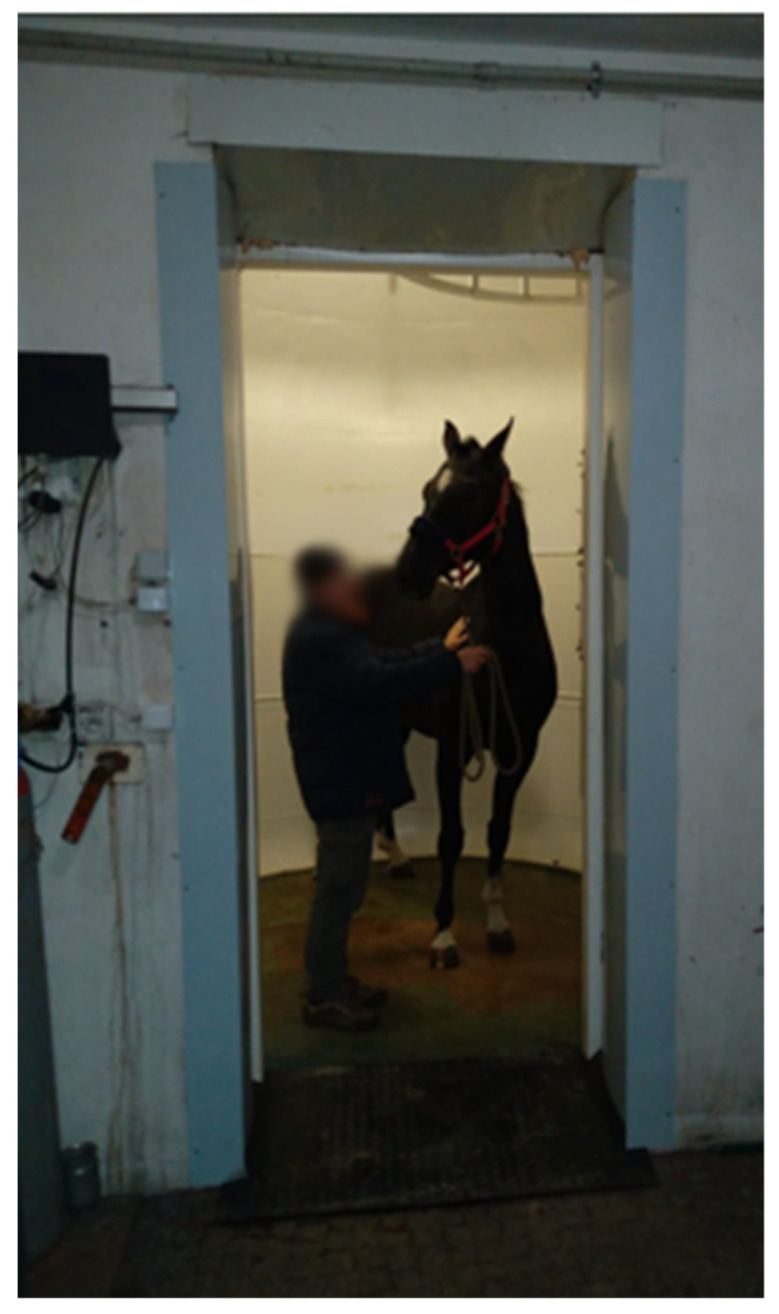
Horse placed in a hyperbaric chamber prior to therapy.

**Figure 14:**
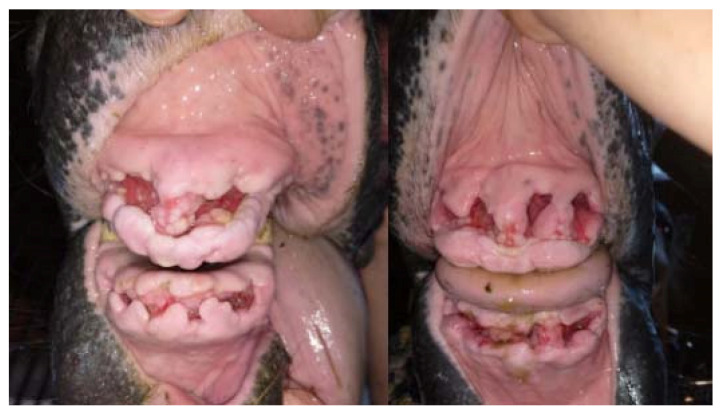
The condition of the teeth pockets 15 days after the procedure (on the left—case 1, on the right—case 2).

The extraction was performed on both horses using the same technique on the same day. It is important that the horses, both before and after the treatment, were kept in the same conditions, and were kept and fed in the same way. Both horses received intravenous flunixin meglumine (1.1 mg/kg body weight) at least one hour before the scheduled surgery. In both cases, premedication was performed with detomidine (20 μg/kg body weight) and intravenous butorphanol (20 μg/kg body weight). A Hausmann speculum was used in order to thoroughly examine the oral cavity again and rinse it with a 0.2% solution of chlorhexidine gluconate. Then, the full mouth speculum was removed. Both maxillary and inferior alveolar nerves were anesthetized by administration of 5 mL of 2% lignocaine hydrochloride to the infraorbital foramen and mental foramina, respectively, and gingival infiltration with 2% lignocaine hydrochloride. Using the horse’s incisor chisel, the gums were pulled off the labial, lateral, and palatal/lingual surfaces. After careful separation of the gums, trying to save their surface as much as possible (this has a positive effect on the subsequent healing process), Liston’s forceps were inserted and clamped between the adjacent teeth, acting as a separator. This resulted in the destruction of the periodontal ligaments, especially on the lateral/medial surface of the tooth. The next step was cutting the alveolar ligaments and separating the teeth from the alveoli. For this purpose, the same chisel was used, and depending on the need, a hammer. After the tooth was partially loosened, it was grasped with the incisor extraction forceps and the tooth was completely loosened and extracted. After extraction of the incisors, the sockets were revised to prevent tooth fragments and damaged lamina dura fragments from being left within them. The oral cavity and alveoli area were rinsed and secured with swabs impregnated with 10% povidone iodine with iodoform. Bandages were placed inside the remnants of the alveolar processes. After the procedure, systemic antibiotic therapy in the form of dihydrostreptomycin (8000 IU/kg b.w.) with procaine penicillin (8 mg/kg b.w.) was administered and was continued for the next 6 days. Analgesic and anti-inflammatory therapy was provided during the first two days after the procedure with phenylbutazone (2.2 mg / kg b.w.) administered orally. The first alveolar bandages were changed 4 days after the procedure (Figure 11), the next one after 5 days (Figure 12). In both cases, due to the proper healing process, the successive filling of the alveolar area with granulation tissue and the reduction of their depth, the application of dressings was stopped. Daily gentle mechanical cleaning and rinsing with a large amount of running water under low pressure was performed. It was also recommended to give grass pellet pulps for the next few days and to avoid giving oats and grains. After the procedure, in both cases the general parameters remained within physiological limits. The horses were fed with roughage and concentrated fodder without any problems. In both cases, regular oral checkups were recommended every 6 months. The healing alveolar processes and gingiva were regularly monitored and documented. This did not require sedation, and both horses became familiar with the rinsing process of the oral cavity and alveolar processes area after extraction of the incisors. The total healing process for both horses took approximately 5 weeks. However, limiting the acute inflammatory process and the speed of wound healing was clearly evident in the horse using the hyperbaric chamber (Figure 12, Figure 13 and Figure 14). In both cases, the only complication after extraction of the incisors was the involuntary protrusions of the tongue from the mouth. According to the owner’s observations, the horse in case 2 had improved behavior. In both horses, no loss of body weight or condition was observed after the treatment. Both horses had no problem eating, even fresh grass. It should be noted that both horses were kept in a variable grazing and stable system. Both horses, despite their advanced age, were used for recreational purposes several times a week under the saddle, and their training consisted mainly of off-road rides.

### 2.3. Hyperbaric Oxygen Therapy

A hyperbaric chamber with a diameter of 3 m and a capacity of 40 m^2^ was used in the therapy (Figure 13). The therapy included the supply of 40% oxygen, 2% carbon dioxide and 0.5% hydrogen at a pressure of 1500 hPa for 90 min. The use of the chamber by the horse from case 1 took place daily from the day of surgery for 25 days. The horse was not previously prepared or trained to stay in the chamber. The horse was brought inside the chamber and remained without sedation. The conditions in the chamber reflected the stall environment, the horse did not need to be tied down and could move freely inside. During therapy, however, the horse did not have access to water and food.

## 3. Discussion

Hyperbaric oxygen therapy is defined as inhalation of oxygen at increased pressure, for a potentially therapeutic benefit in a variety of clinical situations [6]. This type of therapy supports healing and reduces the normal recovery time by helping to transport oxygen to the body’s tissues [7]. The procedure consists of administering pure oxygen to the patient under conditions of increased pressure (2–3 times higher than atmospheric pressure), often in a series of repeated treatments [7]. Henry’s law states that when the partial pressure of oxygen in the inhaled air is increased, the amount dissolved in the blood also increases proportionally. Higher oxygen concentration in blood allows for its easier diffusion to deeper tissues. It promotes angiogenesis (neovascularization), fibroblast growth and increased collagen formation, re-epithelialization, as well as reduced tissue oedema [5]. The higher level of oxygen achieved in the tissues helps kill anaerobic bacteria, even despite poor vascularity. Additionally, it leads to better mechanisms of the aerobic action of neutrophils, and as a result, supports fighting infection and tissue healing [11]. It can be used as a primary therapy or as an adjunct therapy [3].

The use of HBOT has various clinical applications in human medicine [6,7]. This therapy is used, among others, in clinical toxicology (carbon monoxide and cyanide poisoning), difficult-to-heal wounds (diabetic ulcers, crushed wounds, compromised skin grafts), chronic anaerobic infections (actinomyces), necrotizing soft tissue infections, and gas gangrene, infections in the bone [2,4,12,13,14,15,16]. Many different applications of HBOT have also been described in human dentistry. Among them, the most frequently mentioned is preventing the development of radionecrosis by supporting the components of the healing process, as well as improving oxygenation of even the poorly supplied tissues [17]. Additionally, HBOT can be helpful in reducing both pain and swelling in mandibular osteomyelitis associated with areas of pus formation, fistulas, and bone sequestration. In the course of chronic periodontitis, it has been proven to reduce pockets and inhibit the growth of subgingival anaerobes [7]. HBOT is recommended as a method that prepares and supports the process of osseointegration, important for dental implant placement. The action of reducing the inflammation accompanying oral submucous fibrosis and its use as a factor promoting epithelialization in mandible reconstruction have also been described [18].

Its use has so far not been widely described in veterinary medicine, in particular in the treatment of horses. Despite the fact that there are centers in the world where this type of therapy is used in equine medicine, mainly in rehabilitation and treatment of wounds, there are not many scientifically documented cases of the effectiveness of this therapy. Clinical application in horses is based on extrapolation from human and animal research, from human clinical experiences, and from anecdotal clinical experiences and outcome in horses [3]. There are described uses of HBOT as adjunctive therapy in fungal pneumonia, thermal burns, carbon monoxide poisoning, smoke inhalation, closed head injuries, ileus, central nervous system edema (perinatal asphyxia), peripheral neuropathies, sports injuries (exertional rhabdomyolysis), cellulitis, compartment syndrome, ischemic injuries (laminitis), and myositis [1,19,20]. The problem is the availability of this type of chamber for horses, which only a few centers in the world have. In the described cases, the chamber belonged to private owners, thus, cost estimation for commercial use in the course of this disease remains difficult. Our main goal, however, was to document the possibilities offered by the use of a hyperbaric chamber in supporting wound healing in horses, including dentistry, and to show other possibilities of its use.

There is no description of potential complications of this therapy in horses in the literature. In human medicine, complications can be divided into three groups: complications related to oxygen toxicity, complications related to barotrauma, and eye complications [15]. The pulmonary toxicity of oxygen is related to the long-term exposure of the body to 100% oxygen under normal and elevated pressure conditions. Symptoms of oxygen poisoning are irritation of the larynx and trachea, nasal mucosa edema, and periodic pain in the larynx. The risk increases with the frequency of exposure, the duration of exposure, and the number of atmospheres used. Intermittent exposure is helpful in preventing complications. In our case, we used an oxygen concentration lower than 100% to prevent drying of the mucous membranes and a relatively low value of atmospheres (1.4 atmospheres is considered the lowest effective amount [16]). Barotrauma to the middle ear is the most commonly described complication of HBOT in humans. In horses, this problem does not seem to be of great importance due to the presence of guttural pouches in this species, whose role is to equalize the pressure [21]. In humans, rare ocular complications that may occur include transient myopia and cataracts. The reasons for their formation are not well-understood [15].

**Figure 15:**
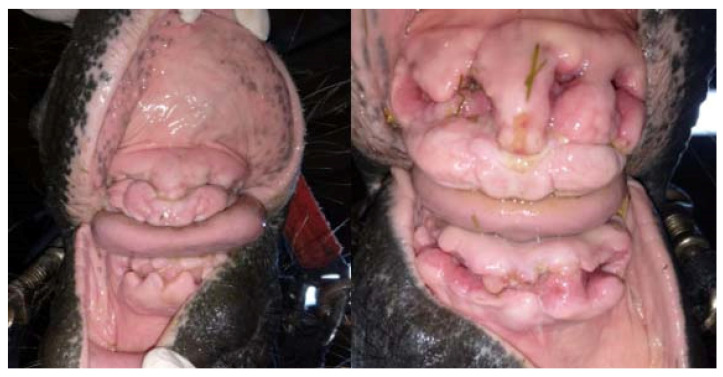
The condition of the teeth pockets 20 days after the procedure (on the left—case 1, on the right—case 2). In a horse not subjected to hyperbaric oxygen therapy (case 2), there is a visible difference in the healing process—the alveolus are much deeper, the gums are less shrunken, and the erythema is still visible.

The article describes the use of this method as a supportive treatment, which accelerates the recovery of the alveoli and gingivae after extraction of incisors in the equine odontoclastic tooth resorption and hypercementosis (EOTRH) syndrome. Its purpose was to determine whether the use of the hyperic chamber has an impact on the wound healing process of incisor extraction. According to the author’s knowledge, this is the first described case of using this type of treatment in equine dentistry. The described cases are a pilot study, and a limitation of the study is the small population of included horses. In order to determine the effectiveness of the therapy using this chamber, a larger group of tested horses should be included, and for the purpose of comparison, a measurement method should be found. EOTRH is a pain-causing condition, that most often affects older horses (over 15 years of age), significantly reducing their quality of life [22,23]. Due to difficulties with food intake, it leads to progressive cachexia. Two separate, coexisting cellular processes play an important role in the disease progression—odontoclastic tooth resorption and hypercementosis [10]. The clinical signs of disease may differ significantly from individual to individual, depending on the dominant process [24,25]. The changes are accompanied by periodontitis, gingivitis, gingival recession, alveolar bone protrusion, periodontic fistulas and soreness of incisors [9]. In the described case, the clinical picture of the disease was the same, and the severity of symptoms was similar, but slightly more advanced in case 1. There are many different theories as to the causes of the disease, but its etiopathogenesis has not been clearly elucidated [9]. The theory of the mechanical stress on periodontal ligaments in older horses seems to be the most probable [26,27]. The formation of mechanical loads on the aforementioned ligaments during prehension causes microtrauma and triggers the inflammatory process. During this process, cementoblasts may be activated, which cause excessive cement deposition. Anaerobic bacteria such as *Treponema spp*. and *Tanerella spp*. have also been isolated from the oral cavity of affected horses, so their contribution to disease triggering is also taken into account [28]. The treatment of choice (and the only effective treatment method) is extraction of the affected teeth, which remarkably diminishes the chronic pain and other symptoms connected with this disease. The total time until the teeth sockets are filled with granulation tissue, depending on their size, is about 6 weeks [9]. Wound healing can be influenced by many different factors, such as nutritional status and coexisting diseases [29]. In the described case, the horses were kept in the same conditions, and were fed and used in the same way. The healing process can also be influenced by the age of the horse (the size of the alveolus after extraction depends on the length of the reserve crown), thus the advantage of this comparison is the fact that both horses are of similar age. Nevertheless, it may be a limitation that the wound healing process may be also influenced by individual factors. Although the quality of life of horses ultimately improves significantly when fully healed, the healing time is associated with discomfort. Many owners decide not to undergo the procedure due to its invasiveness and the vision of a relatively long healing time. The methods of assisting the healing process may shorten the recovery time. In the case of EOTRH disease, the positive effect of the use of hyperbaric oxygen therapy may result not only from supporting the wound healing process (including the stimulation of angiogenesis, supporting the mechanisms of aerobic inflammatory cells, stimulation of the granulation process), but also effective action on anaerobic bacteria, which are believed to play a significant role in this disease. In the authors’ opinion, the healing process was similar in both horses. However, the reduction of the inflammatory process appeared to occur more quickly in the treated horse (case 1). Compared to case 2, the gingival contraction process associated with wound healing was noticeably more dynamic in case 1 (Figure 14 and Figure 15).

## 4. Conclusions

Although in the cases described, the healing of the wound would occur spontaneously following extraction of the incisors without the aid of the chamber, the presented case allows for the comparison of the healing rate of the extraction wound with and without application of the chamber. Additionally, during the conducted observations, no side effects of the therapy were reported. Further observations should concern the use of the hyperbaric chamber in a larger group of horses. Comparing the effectiveness of treatment, as well as parameters used during the therapy and diseases in which it can be used, would increase the knowledge on the effectiveness of its application.

## Data Availability

The data presented in this study are available in the manuscript.
